# A case of congenital single testis with testicular cancer patient and azoospermia who was able to collect spermatozoa with ipsilateral Onco‐TESE

**DOI:** 10.1002/ccr3.3576

**Published:** 2020-11-23

**Authors:** Kazumasa Hayashi, Teruo Inamoto, Haruhito Azuma, Hiroshi Masuda, Hirotsugu Oku

**Affiliations:** ^1^ Department of Urology Ishinkai Yao Sogo Byoin Yao Japan; ^2^ Department of Urology Osaka Medical College Takatsuki Japan; ^3^ Department of Urology Tesseikai Neurosurgical Hospital Shijyounawate Japan; ^4^ Department of Urology Ladies Clinic Kitahama Osaka Japan

**Keywords:** cancer patient, congenital single testis, Onco‐TESE, testicular cancer

## Abstract

Onco‐TESE is a useful strategy for cancer patients with a congenital single testis who wish to preserve their fertility.

## Graphical abstract

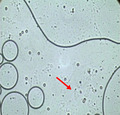

## 1 INTRODUCTION

A young Japanese man presented with testicular cancer in a congenital single testis and was subsequently diagnosed with azoospermia. The patient underwent orchidectomy and simultaneous ipsilateral oncological‐testicular sperm extraction, which collected viable sperm for cryopreservation. The sperm was used for intracytoplasmic sperm injection, and a healthy child was ultimately born.

Testicular germ cell tumors (TGCTs) are most commonly found in men who are 15‐35 years old and account for 1.0‐1.5% of neoplasms in men.^1^ Men have also had an increasing incidence of testicular dysgenesis syndrome (TDS, decreased spermatogenesis, hypospadias, cryptorchidism, and testicular tumors) during recent decades, and the four events are often interrelated.^2^ Patients with TGCT can typically be cured, although postoperative chemotherapy and/or radiotherapy typically damage the healthy testis and result in infertility. Several researchers have recommended cryopreservation of sperm before treatment for TGCT,^3^, ^4^, ^5^ as infertility can harm the mental health of young patients, and these patients may not have considered their fertility or plans to have children at the time of their TGCT diagnosis. Thus, pre‐treatment cryopreservation of sperm may improve these patients' quality of life, even if the sperm is not ultimately used for assisted reproductive therapy.^6^ Furthermore, 75% of these patients have not had children at the time of their diagnosis and >50% of patients with cured testicular tumors may desire a child after treatment.^7^

Although the American Society of Clinical Oncology recommends sperm cryopreservation to preserve fertility in this setting, few patients receive infertility treatment,^8^ and studies at two major cancer centers revealed that only 51% of patients had completed sperm banking and only 24% of patients actually had their sperm cryopreserved.^9^ Nevertheless, patients with TGCT have cure rate of 80‐90% after treatment using surgery, radiation, and chemotherapy, and an increasing trend has been identified,^10^ with strict follow‐up and salvage therapy contributing to good outcomes.^11^, ^12^, ^13^ Onco‐TESE is defined as a testicular sperm extraction in azoospermic cancer patients before chemotherapy that allows spermatozoa to be obtained from the normal testis of patients who do not emit sperm prior to cancer therapy to preserve and treat fertility in these men at the time of their orchiectomy. Therefore, oncologic testicular sperm extraction (Onco‐TESE) might have value for preserving fertility among patients with TGCTs and azoospermia, as sperm that are identified in non‐cancer tissues can be cryopreserved and subsequently used for assisted reproductive therapy. We describe our experience with a patient who had testicular cancer in a congenital single testis and was subsequently diagnosed with azoospermia. Ipsilateral Onco‐TESE was performed simultaneously during radical orchiectomy and sperm was collected, cryopreserved, and subsequently used for intracytoplasmic sperm injection (ICSI). The patient's wife ultimately gave birth to a healthy child.

## 2 CASE PRESENTATION

A 26‐year‐old Japanese man noticed painless enlargement of his right scrotum and visited the urology department at a nearby hospital. He had been diagnosed with cryptorchidism during childhood and had undergone laparotomy, which failed to identify his left testis and he was subsequently diagnosed with a left testicle defect. At his presentation, the right testis was swollen to a diameter of 5 cm and ultrasonography confirmed a 5 cm hypoechoic mass in the right testis. Plain computed tomography revealed a uniform approximately 5 cm mass inside the scrotum with no obvious metastasis. Laboratory tests revealed high concentrations of human chorionic gonadotropin (HCG: 16.6 mIU/mL) and human chorionic gonadotropin‐β (HCG‐β: 0.46 ng/mL), with nearly normal results for α‐fetoprotein (2.3 ng/mL), luteinizing hormone (2.1 mIU/mL), follicle‐stimulating hormone (4.7 mIU/mL), and testosterone (470 ng/dL).

**FIGURE 1:**
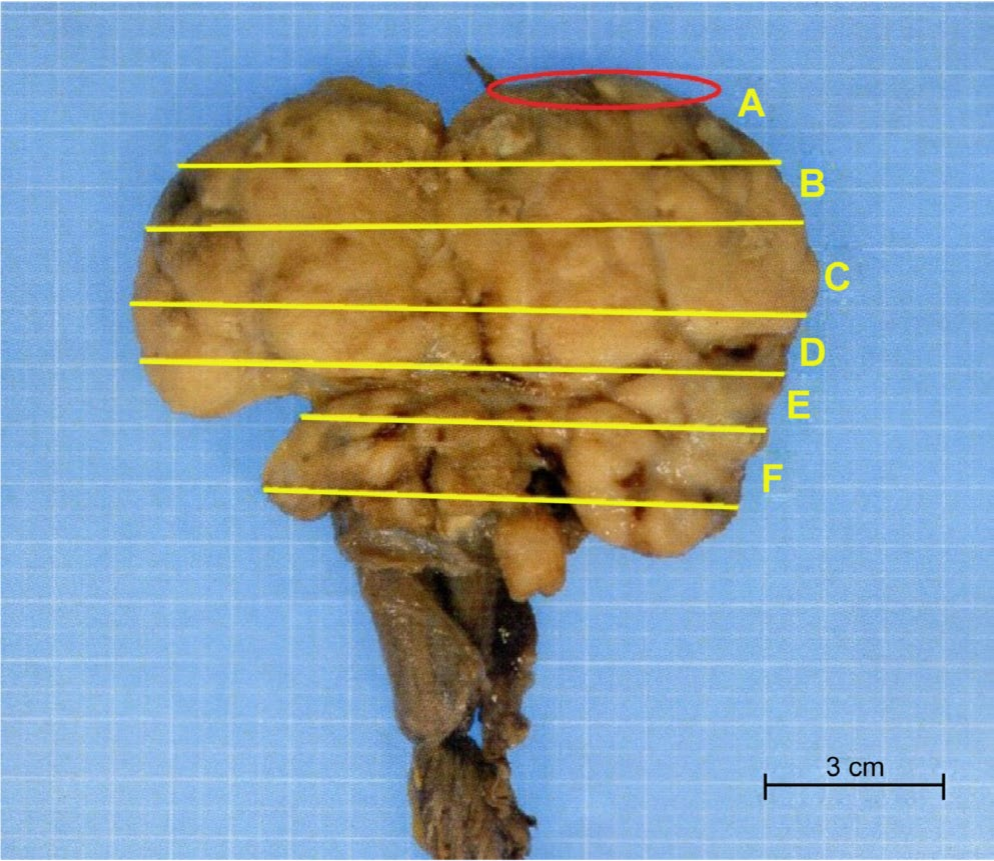
Most of the testis was occupied by the tumor. The seminiferous tubule was excluded by the tumor and clung to the tunica albuginea (near the red circle)

**FIGURE 2:**
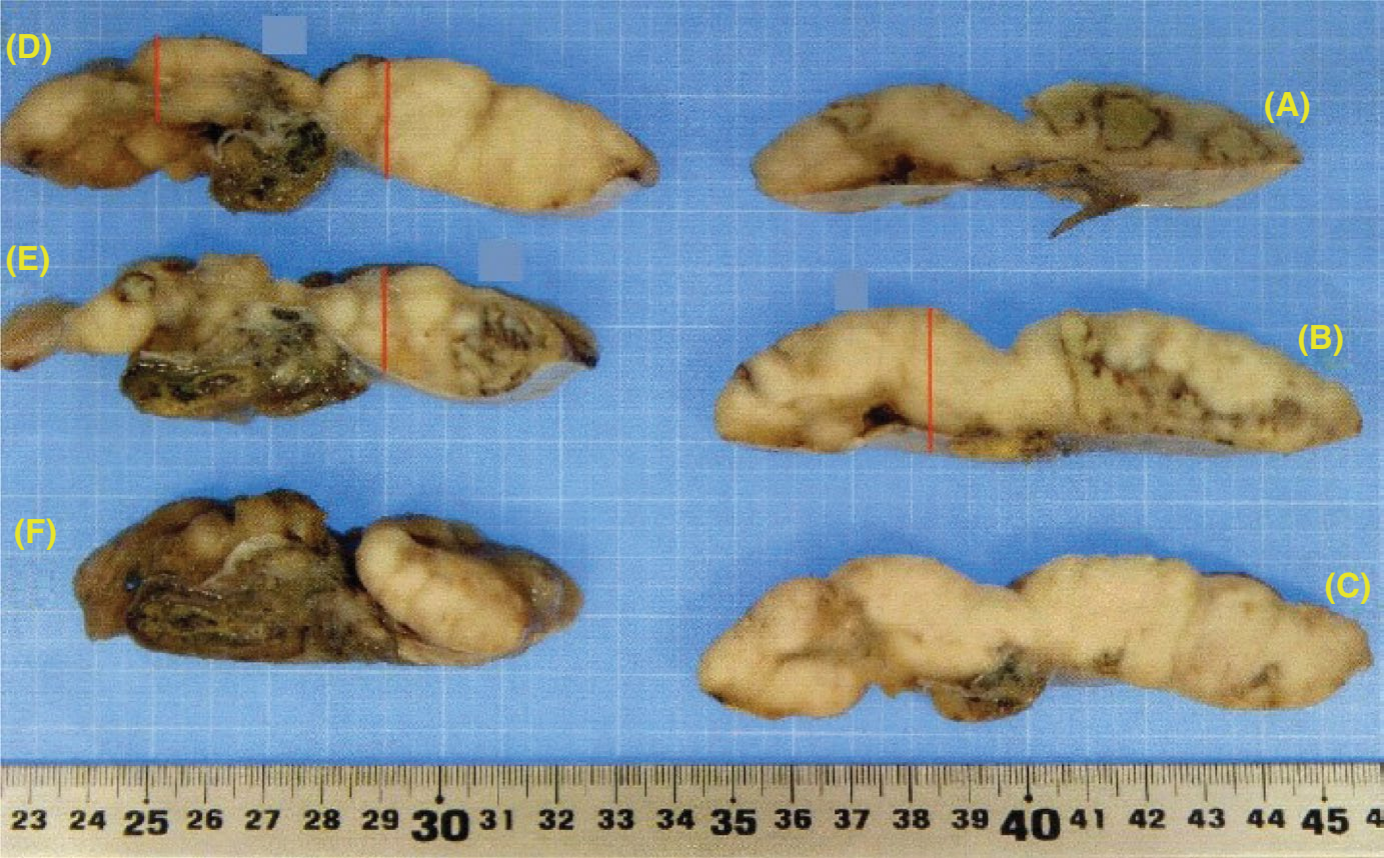
Cut surfaces of the tumor. The red line is the one used for histopathological examination

**FIGURE 3:**
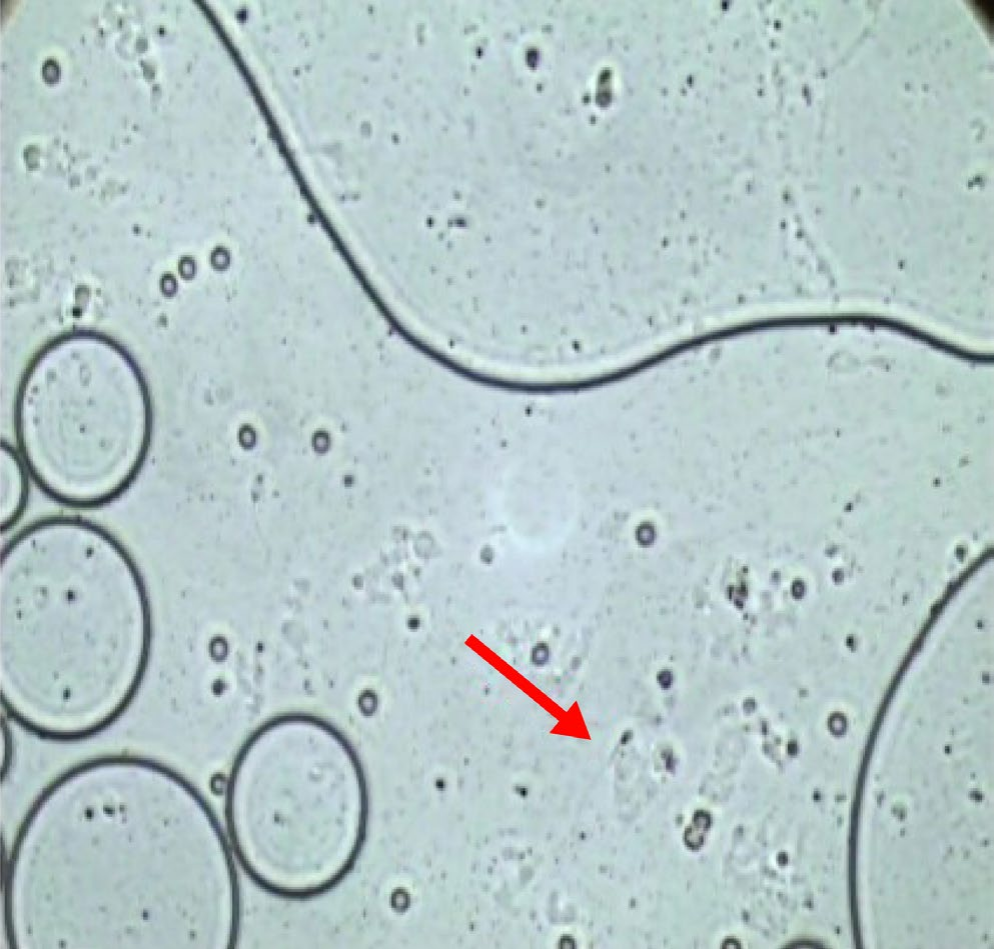
Sperm could be identified using an inverted microscope (×1000)

The patient had a fiancé and wished to cryopreserve his sperm, although azoospermia was identified based on twice semen tests that were performed at the assisted reproductive therapy clinic. His karyotype and Y chromosome were normal and without microdeletions. He was referred to our hospital and underwent radical orchiectomy with planned sampling of any non‐cancer seminiferous tubule tissue to collect sperm if possible. The orchiectomy revealed that most of the testicle was occupied by a gray‐white mass without bleeding or necrosis (Figures 1 and 2), and the seminiferous tubule was visible to the naked eye between the tumor's head and the tunica albuginea. Thus, immediately after testis removal, we collected the seminiferous tubules in situ. We used an open technique that removes several small part of testicular tissue. Samples contained a large amount of cellular debris, including blood clots. Finding sperm in the testicular tissue took about 3 hours to process. The testicular tissue was evaluated under a stereomicroscope to identify seminiferous tubules and remove blood clots. Distribution and localization of seminiferous tubules containing spermatogenesis among the normal tissue were in diffuse fashion. Following identification, the testicular sample was processed by dispersion of the tubules through mechanical homogenization. Thereafter, the sample was evaluated using the inverted microscope (400× magnification) to identify the presence of morphologically normal and motile sperm (Figure 3). Approximately 0.5 g of tissue was collected, divided into 10 tubes, and we have sent the rest of resected testicular tissue to the cryopreservation laboratory. The excised testicular tumor was about 12 cm in diameter.

**FIGURE 4:**
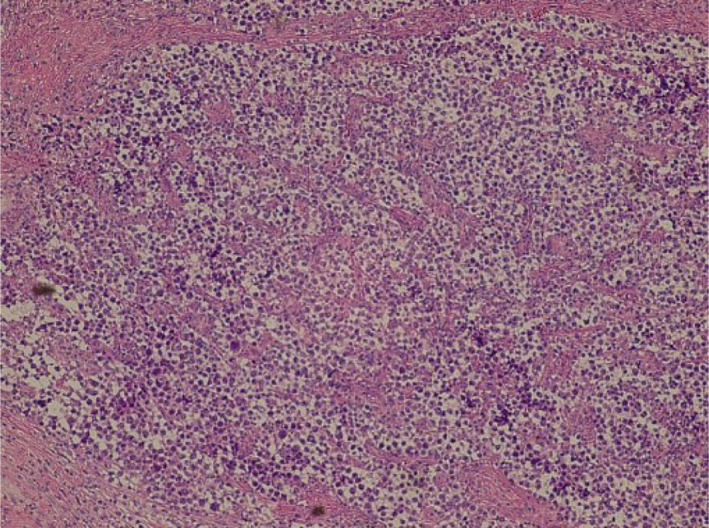
Hematoxylin and eosin staining (×400) revealed tumor cells containing lymphocytes and clear cytoplasm

**FIGURE 5:**
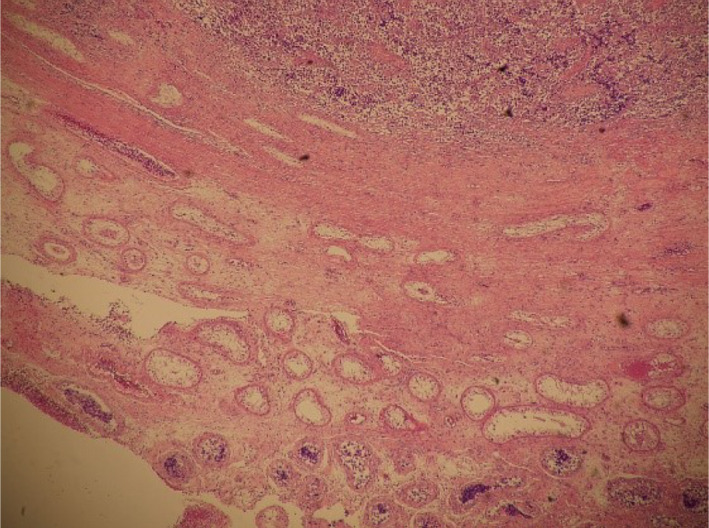
Seminiferous tubules were observed between the head of the tumor and the tunica albuginea (× 100)

Histopathological examination revealed tumor cells that contained lymphocytes and clear cytoplasm, which supported a diagnosis of pure seminoma (pT1N0M0) (Figure 4). Spermatogenesis was confirmed in a portion of the normal seminiferous tubules (Figure 5). The seminiferous tubules had uniform thickness throughout and diffuse spermatogenesis. The Johnson score was 8.

The patient's postoperative course was uneventful, with normalization of his HCG and HCG‐β concentrations. No adjuvant therapy was performed, although close follow‐up was performed using imaging and blood tests. No obvious tumor recurrence has been observed, and the patient is receiving continuous testosterone supplementation because of low testosterone concentrations after the orchiectomy. The patient subsequently married his fiancé, who had normal gynecological findings based on hysteroscopy, transvaginal ultrasonography, and blood tests. Thus, ICSI was performed using the cryopreserved sperm, the wife became pregnant, and a healthy boy was born on gestational week 39 + 3 (birth weight: 3,060 g, no TDS syndrome).

## 3 DISCUSSION

In this case, the patient was preoperatively diagnosed with azoospermia, and onco‐TESE was the only option to preserve his fertility because the congenital single testis needed to be removed. Testicular tumors are generally associated with decreased spermatogenesis, with patients having approximately 33% of the sperm concentration and 17% of the total sperm count, relative to men in the general population.^14^ The effects on spermatogenesis are pronounced after chemotherapy, although recovered spermatogenesis has been identified in 75% of patients after 18 months.^15^ However, this would not have been possible in our case, given the removal of his congenital single testis.

The reported frequency of congenital single testis diagnosed during surgery for cryptorchidism is 4% in Europe and the United States.^16^ However, assisted reproductive technology has improved in recent years, and these patients may be able to preserve their fertility using cryopreserved sperm.^17^, ^18^ Several groups^6^, ^19^, ^20^, ^21^ have reported that Onco‐TESE is possible in patients with azoospermia and testicular cancer, which may allow the cryopreserved sperm to be used with assisted reproduction to achieve the birth of a healthy baby. However, we are not aware of any reports that have described this strategy in a patient with testicular cancer involving a congenital single testis.

## 4 CONCLUSION

We performed Onco‐TESE during radical orchiectomy and were able to preserve the fertility of a patient with a congenital single testis. This technique successfully retrieved sperm that were cryopreserved and used for ICSI, which ultimately resulted in the birth of a healthy child. Therefore, Onco‐TESE is a useful strategy for cancer patients with a congenital single testis who wish to preserve their fertility.

## CONFLICT OF INTEREST

None declared.

## AUTHOR CONTRIBUTIONS

All authors: read and reviewed the manuscript. HM: performed the orchidectomy, Onco‐TESE, and postoperative follow‐up. TI and HA: reviewed the manuscript. HO: referred the patient to our clinic and successfully performed ICSI with the cryopreserved sperm.

## Data Availability

The data that support the findings of this study are available on request from the corresponding author. The data are not publicly available due to privacy or ethical restrictions.
